# Cascade of Inflammatory, Fibrotic Processes, and Stress-Induced Senescence in Chronic GVHD-Related Dry Eye Disease

**DOI:** 10.3390/ijms22116114

**Published:** 2021-06-06

**Authors:** Yoko Ogawa, Yutaka Kawakami, Kazuo Tsubota

**Affiliations:** 1 Department of Ophthalmology, Keio University School of Medicine, Tokyo 160-8582, Japan; tsubota@z3.keio.jp; 2 Division of Cellular Signaling, Institute for Advanced Medical Research, Keio University School of Medicine, Tokyo 160-8582, Japan; yutakawa@keio.jp; 3 Department of Immunology, School of Medicine, International University of Health and Welfare, Chiba 286-8686, Japan

**Keywords:** dry eye disease, graft-versus-host disease, molecular mechanism, stress-induced senescence

## Abstract

Ocular graft-versus-host disease (GVHD) is a major complication after allogeneic hematopoietic stem cell transplantation. Ocular GVHD affects recipients’ visual function and quality of life. Recent advanced research in this area has gradually attracted attention from a wide range of physicians and ophthalmologists. This review highlights the mechanism of immune processes and the molecular mechanism, including several inflammation cascades, pathogenic fibrosis, and stress-induced senescence related to ocular GVHD, in basic spectrum topics in this area. How the disease develops and what kinds of cells participate in ocular GVHD are discussed. Although the classical immune process is a main pathological pathway in this disease, senescence-associated changes in immune cells and stem cells may also drive this disease. The DNA damage response, p16/p21, and the expression of markers associated with the senescence-associated secretory phenotype (SASP) are seen in ocular tissue in GVHD. Macrophages, T cells, and mesenchymal cells from donors or recipients that increasingly infiltrate the ocular surface serve as the source of increased secretion of IL-6, which is a major SASP driver. Agents capable of reversing the changes, including senolytic reagents or those that can suppress the SASP seen in GVHD, provide new potential targets for the treatment of GVHD. Creating innovative therapies for ocular GVHD is necessary to treat this intractable ocular disease.

## 1. Introduction

Allogeneic hematopoietic stem cell transplantation (HSCT) is now an established treatment for hematological malignancies and other life-threatening hematological and genetic diseases [1]. The major barrier to successful HSCT is graft-versus-host disease (GVHD), which is one of the most frequent complications after HSCT [2]. The eye is a frequently affected organ after HSCT, and complications mostly present as dry eye disease [3,4,5,6]. More than 50% of recipients develop dry eye disease approximately 6–24 months after HSCT [7,8]. Severe GVHD-related dry eye disease can progress rapidly and lead to blindness, and even in less severe cases, it has a significant impact on patients’ quality of life and quality of vision because humans can obtain 70–80% of their sensory information from vision. Previous acute GVHD, disparity of HLA or MHC or minor histocompatibility antigens, aged donor or recipient, the presence of diabetes, stem cell source of transplantation, female to male transplantation, and Epstein–Barr Virus seropositive donor are reported as risk factors for developing ocular GVHD [6].

The ocular surface consists of a tear film layer, the lacrimal glands, accessory glands, the cornea, conjunctiva including goblet cells [9], the nasolacrimal ducts, the meibomian glands, and possibly glands of Zeis because hair follicles are commonly targeted in skin GVHD [10]; these components maintain the homeostasis of the ocular surface and protect the eye from invading pathogens and other environmental challenges. Ocular surface immunity involves communication between local and systemic inflammatory cells and interstitial stromal cells or epithelial cells by interactions mediated by specific cell surface receptors and soluble factors [11]. The ocular surface becomes an immune-advanced site once homeostasis is dysregulated by a disturbance in the microenvironment on the ocular surface including the goblet cell, which have immunomodulatory function besides mucin production [12]. Goblet cells play important roles for ocular surface health, maintaining dendritic cells in an immature state [13], reducing the number of those cells that are reported to be linked to various inflammation pathways by changing acting antigen-presenting cells [14], and regulating inflammatory cytokines, including IL-13 [15] or interferon-γ [16]. Eye-associated lymphoid tissue (EALT) has been proposed, which is continuous at the ocular surface, including the cornea, conjunctiva, and meibomian gland and its adnexa, including the lacrimal glands (LGs) and lacrimal drainage system [11]. A breakdown of EALT homeostasis may result in dry eye disease [17]. This concept is likely to be associated with immune-mediated dry eye disease, including GVHD, Sjögren’s syndrome, and other conditions of dry eye associated with autoimmune diseases [18]. Specifically, ocular GVHD (oGVHD), representing as dry eye disease, is characterized by chronic inflammation and pathogenic fibrosis on the ocular surface, resembling ocular cicatricial pemphigoid and Stevens–Johnson syndrome [19]. In this review, altered mucosal immunity, pathogenic fibrosis, and stress-induced senescence in oGVHD are discussed. By focusing on the eye region, those findings may be linked to better understanding in other targeted organs in GVHD and other inflammatory fibrotic diseases and age-related diseases and assist the development of novel and innovative therapy in the future. 

### 1.1. Mucosal Barrier of the Ocular Surface

The mucosal immune system includes dendritic cells, macrophages, mast cells, lymphocytes, fibroblasts, and soluble factors interacting with the surface barrier epithelium, including goblet cells and microbiota that collectively contribute to immune surveillance [20]. The conjunctiva has several defense systems that protect the integrity of the surface epithelium and associated mucin layer, including antimicrobial peptides and secreted IgA, which adhere to the mucin layer. The role of IgA has been elucidated in some detail. IgA-producing plasma cells reside in the subepithelial stroma of the lacrimal glands and in the ocular surface mucosal membrane. Secreted IgA dimers bind the IgA receptor on the basement membrane of the basal epithelium and are taken up, transported intracellularly, and thereafter excreted into the tear film by the ocular surface epithelium along with other secretory components [21]. When ocular immune homeostasis is disrupted, the integrity of the ocular surface can break down, resulting in inflammatory mucosal disease and dry eye disease [17]. In ocular GVHD, basement membranes of conjunctival and lacrimal gland epithelia are disrupted through being targeted by T cells [22,23], resulting in an altered balance of production of IgA or the secretion of secretory IgA on the ocular surface. 

#### Tear Film in GVHD-Related Dry Eye Disease

Tear fluid consists of a unique structure containing lipids, water, mucins, a variety of proteins, and electrolytes [24]; it plays a crucial role in visual function, lubrication, protection from invading foreign bodies and pathogens, washing out hazardous materials, and supplying moisture, nutrition, and oxygen on the ocular surface [24]. It is a mysterious fluid found only on the ocular surface. Certain tear cytokines have been proposed as possible biomarkers for ocular GVHD due to elevated interferon-γ, intercellular adhesion molecule-1, interleukin (IL)-6, IL-8, IL-1β, IL-10, IL-17, CXCL-10, tumor necrosis factor (TNF)-α, soluble TNF -α receptor 1, and neutrophil elastase, [25,26,27,28,29,30], MMP-9,VEGF [27], extra DNA [31] and anti-citrullinated protein autoantibodies [32] in the tear film. The level of neutrophil elastase is reported to be correlated with the elevation of myeloperoxidase, MMP8 and MMP9, suggesting the substantial role of neutrophils in tear film in ocular GVHD [33]. Those findings suggest that doxycycline and tissue inhibitors of metalloproteinases (TIMPs) are potentially reducing neutrophils and MMPs and are useful as therapeutic targets in ocular GVHD. It has been suggested that IL-8/CXCL8 and IP-10/CXCL10 have optimal sensitivity and specificity for indicating ocular GVHD [34]. Proteome analyses revealed elevated cytoskeleton protein and nuclear acid binding protein [35]. Decreased levels of IL-7, IP-10, EGF [28], lactoferrin [36], lactotransferrin, transfer and receptor proteins, enzyme modulators, and hydrolases [35] in ocular GVHD patients are reported, suggesting the loss of those regulatory factors for defending pathogens or foreign bodies from the outside world in the ocular surface in patients with cGVHD. Further elucidation of the function and cellular origin producing each soluble mediator is necessary for understanding the underlying mechanism of pathophysiological processes and improving the ocular surface condition in GVHD-related dry eye disease.

### 1.2. Microbiota

The commensal microbiota coevolves with the host in a symbiotic relationship on the ocular surface [37,38]. The host microbiota possesses multiple abilities to activate immunity and contribute to the deterioration of ocular surface tissue related to infections and immune-mediated diseases including GVHD [39,40]. HSCT patients treated with irradiation, immunosuppressive reagents, and/or corticosteroid therapies may lead to immune-compromised conditions in the mucosal immune system. Those factors may lead to altered and pathogenic changes of the microorganisms on the ocular surface. Several reports on the ocular surface show significantly diversified microbiota in GVHD and Stevens–Johnson syndrome using conventional cultures [37] and next-generation sequencing methods [40,41], and they also show characteristic elevation of Lactobacillus and α-diversity in ocular GVHD [41]. In contrast, several studies show dysbiosis in several immune-mediated disorders including Sjogren’s syndrome using 16S ribosomal RNA gene sequencing [39]. There is a close relationship between systemic GVHD and the gut microbiome showing loss of diversity in the intestinal microbiota of HSCT recipients. 

In ocular GVHD, the initial site of ocular GVHD might be conjunctival epithelium including goblet cells [12,13,14,15,16], main excretory ducts of conjunctiva, lacrimal glands, and meibomian glands [7,9,22,42,43,44,45,46,47,48,49]. An infiltration of various inflammatory cells in the epithelial region of conjunctiva and main exocrine ducts with goblet cells and capillaries primarily via interactions with T cells, macrophages, and fibroblasts is observed in patients with cGVHD.

An obviously reduced number of goblet cells in ocular GVHD [9,49,50] is related to dysregulated immune function, leading to severe and irreversible damage in ocular GVHD based on the previous reports [12,13,14,15,16,22,48]. The lumen of lacrimal gland and meibomian gland ducts as well as ocular surface including cornea and conjunctiva faces the outside world, and commensal microbiota exist on the mucosal membrane of the ducts. These findings suggest that the interaction of mibrobiota and the mucosal immunity on the ocular surface of the duct lumen is potentially triggering the ocular GVHD. Further elucidation will be needed regarding the relation between the decreased diversity of microbiota in the gastrointestinal tract and elevated diversity of ocular surface in ocular GVHD.

## 2. Ocular Surface Inflammation

### 2.1. Early Phase of Inflammatory Response

GVHD is triggered by the interaction of migrating T cells and antigen-presenting cells [51]. Th1 and Th 17 differentiation with decreased regulatory T cells amplifies APC activation, resulting in a cytokine storm. Intensive chemotherapy or irradiation, as a preparative regimen before HSCT, may affect the ocular surface barrier of the conjunctival epithelium and facilitate the leakage of altered microbial metabolites and damage-associated molecules into the subconjunctival stroma and peripheral blood [1]. The disruption of conjunctival mucosal barrier and damage is associated with ocular GVHD, as shown in other mucosal membrane in target organs of GVHD [52]. In ocular GVHD, pseudomembranes of tarsal conjunctiva are frequently observed [53,54,55]. The histopathology of the pseudomembrane is reported as an infiltration of mature T cells from the donor graft to the recipient’s conjunctival epithelia. The pseudomembrane consists of damaged epithelia with a large number of macrophages [53,54,55,56,57,58,59]. Macrophages infiltrate as scavengers to phagocyte the degenerated epithelia as a damage-associated molecular pattern. Plenty of macrophages, CD8^+^ T cells, CD4^+^ T cells, and fibroblastic cells are reported to concomitantly infiltrate into the degenerated epithelia, suggesting an overlap syndrome of acute and chronic GVHD in the ocular region [55,56], leading to cytokine storm [51,60], which leads to corneal ulcer [55]. Pathological fibrosis presumably developed by chemokines such as stromal-derived factor-1 stimulates the chemotaxis of the fibroblasts. 

Studies have shown that type 2 innate lymphoid cells (ILC2s) recruit myeloid-derived suppressor cells that suppress T cell-mediated GVHD and ILC3s secreting IL-22, enhancing intestinal stem cell function, promoting repair and the release of adenosine and suppressing T cell proliferation [61,62,63,64,65]. It is likely that an altered or reduced number of ILCs play some role under allogeneic circumstances of the ocular surface of dry eye associated with cGVHD. Further studies are required to clarify those issues.

The development of ocular GVHD is a multistep and multifactorial process. (1) A variety of inflammatory cells, including macrophages, dendritic cells, natural killer cells, in innate immunity and T cells, and B cells in adaptive immunity; (2) conjunctival, lacrimal gland, and meibomian gland epithelial cells, including conjunctival goblet cells; and (3) mesenchymal cells all play pivotal roles in the development of ocular GVHD in mice and humans [23,45,49,66,67,68,69,70].

Infiltrated dendritic cells and globular cells increasingly surround the corneal nerves in ocular GVHD recipients compared to non-GVHD recipients [71], while another study showed that those findings are similar in both groups [72]. Confocal microscopic findings can detect morphology and not be able to identify the exact cell type. However, there are two types of cells in the images of corneal confocal microscopy, and dendritic cells might be Langerhans cells and globular cells might be lymphocytes, both of which contribute to the ocular surface inflammation. The Toll-like receptor 2 (TLR2)-nuclear factor kappa-light-chain-enhancer of activated B cells (NF-κB) signaling pathway in patients with dry eye disease associated with cGVHD has been shown to be significantly activated, suggesting that TLR2 has a significant role in the inflammatory milieu of cGVHD-related dry eye disease [73].

In the conjunctiva, squamous metaplasia, decreased mucin production due to decreased goblet cells [50], decreased secretory vesicles, decreased membrane spanning mucins [49], thinning of conjunctival epithelia, and altered epithelial cell morphology have been reported [23] in patients with cGVHD dry eye disease. Under ultrastructural observation, distorted or branched microvilli of conjunctival epithelia and loss of secretion for membrane-spanning mucin can be observed and may lead to severe dry eye disease in mice [45] and humans [49]. Macrophages and T cells including CD4^+^ and CD8^+^ T cells are predominantly infiltrated in the cornea and conjunctiva of animal models of GVHD-related dry eye disease [45,66] and conjunctiva and lacrimal gland of human patients [48,68]. An increase in Th 17 cells and decrease in regulatory T cells by IL-6 production through interactions between T cells and mesenchymal stem cells in the lacrimal gland and conjunctiva may lead to dry eye disease progression in an animal model of GVHD [45,69].

**Figure 1:**
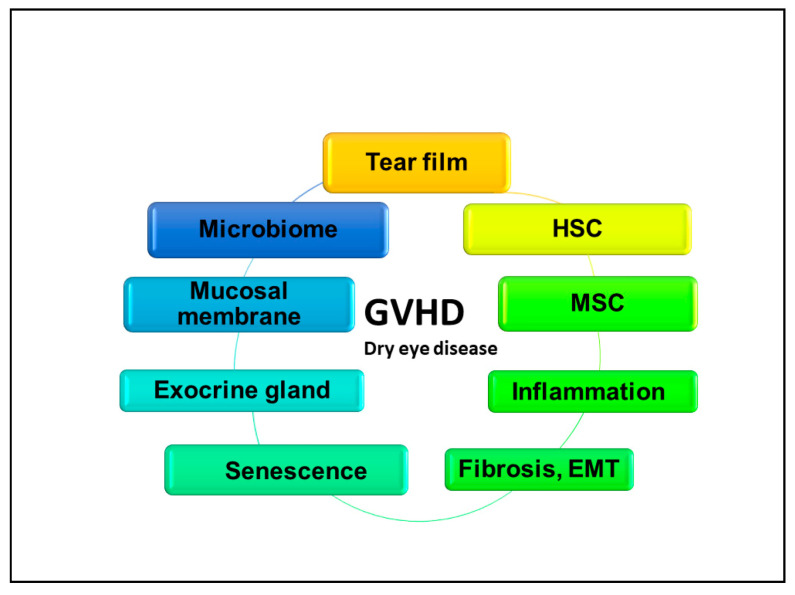
A variety of biological areas orchestrate in ocular GVHD, dry eye disease, which are representative of a manifestation of ocular GVHD and are related to various aspects of the study area including the tear film, microbiome, mucosal and exocrine gland immunity, stem cell biology on HSC or MSCs, acute and chronic inflammation, pathogenic fibrosis including fibroblasts origin, and stress-induced senescence. Those pathways are connected timely and spatially to each other and the development of ocular GVHD, MSC; mesenchymal stem cells, HSC; hematopoietic stem cells, EMT; epithelial mesenchymal transition.

These inflammatory cytokines further amplify pro-inflammatory cytokine and chemokine production and recruit other inflammatory cells, including altered macrophages, mesenchymal cells, including mesenchymal stem cells, fibroblasts, and endothelia or T cells and B cells, which collectively activate the expression of adhesion molecules and conjunctival vascular endothelial molecules [68,69]; these molecules further facilitate the recruitment of inflammatory cells to the ocular surface, thus generating an inflammatory microenvironment [11]. Chronic irritation such as tarsal conjunctival fibrosis or trichiasis with microbial pathogenic change in an immune-compromised host may develop under the influence of one or several triggers, causing chronic inflammation, which can lead to accelerate cGVHD-related dry eye disease (Figure 1).

### 2.2. Chronic Inflammation

Chronic immune inflammation of the eye includes the acquisition and processing of antigens by ocular antigen-presenting cells, which migrate to the draining lymph nodes through conjunctival afferent lymphatics and veins and then prime naïve CD4^+^ T cells and induce Th1 and Th17 polarization [74]. Then, primed CD4^+^ T cells migrate into the conjunctiva and lacrimal gland, where they attach to the activated vascular endothelium and migrate into the targeted microenvironment. Cytokines such as IFN-α and IL-17, which are produced by activated T cells, amplify the immune response by increasing the expression of adhesion molecules including VCAM, VEGF-C, and VEGF-D on conjunctival blood vessels, which leads to ocular surface damage [11,75]. Effector memory Th17 cells contribute to maintain the chronic and relapsing course of dry eye disease [76]. IFN-γ also alters mucins on the ocular surface; these pathogenic changes are implicated in epithelial cell apoptosis, goblet cell loss, and squamous dysplasia [77]. IL-17 increases MMP3 and MMP9 expression and corneal epithelial barrier dysfunction [75]. At the site of corneal perforation, macrophage or CD8^+^ T cell accumulation and MMP9 elevation are observed in GVHD patients [78,79].

Although these changes do not always occur in all stages of cGVHD-related dry eye disease, immune-mediated inflammation, including the dysregulated activation of innate and adaptive immunity, is a pivotal factor in developing and perpetuating this disease. 

### 2.3. Sterile Inflammation

Sterile inflammation, which can be triggered by a wide variety of stimuli, including altered cellular and tissue states or debris, is reported to be associated with GVHD [80]. A body of evidence indicates that extracellular DNA (eDNA), a damage-associated molecular pattern, may be related to cGVHD-related dry eye disease [31,81]. DNase levels are reported to be extremely reduced in the tear film of cGVHD patients [82]. Therefore, eDNA accumulates on the ocular surface, resulting in the severe form of dry eye disease in cGVHD. In addition to macrophage, natural killer cell, T cell, and B cell infiltration, danger signals such as eDNA are reported to be related to the severity and perpetuation of dry eye disease. eDNA is released at the ocular surface by superficial dying conjunctival or corneal epithelial cells, probably resulting in filamentosa on the cornea that leads to severe pain and discomfort in ocular GVHD patients. In patients of cGVHD-related dry eye disease, cell debris in pro-inflammatory state are accumulated in lacrimal gland stroma in humans [83]. With multiple functions in both the innate and adaptive immune systems, autophagy has been demonstrated to take part in the pathogenesis of several immune-related diseases. The study of the pathological mechanism of autophagy may provide a targeted intervention directed against the therapeutic strategy of immune diseases including ocular GVHD.

## 3. Immune-Mediated Fibrosis

Fibrosis is the extensive deposition of fibrous connective tissue, and it is characterized by the accumulation of collagen and other extracellular matrix (ECM) components [84]. Fibrotic remodeling in various diseases, such as liver cirrhosis, pulmonary fibrosis, systemic sclerosis, and GVHD, can lead to organ dysfunction, causing high morbidity and mortality. Both innate immunity and adaptive immunity are involved in fibrogenesis in ocular GVHD [22,84,85]. Published data support the roles of profibrogenic molecules, including IL-4, IL-6, IL-17, heat shock protein 47, connective tissue growth factor, and transforming growth factor (TGF)-β, in fibroblast activation in ocular cicatricial pemphigoid [86,87,88] that presents similar fibrotic conditions of ocular GVHD. Novel therapeutic options based on inhibiting these pathways will be required.

### 3.1. Bone Marrow-Derived Cells in Pathogenic Fibrosis

Stromal mesenchymal cells, such as fibroblasts, are classically thought to produce collagens to simply support various organs, but a body of evidence indicates that these mesenchymal cells play some role in immunosurveillance or act as immune cells themselves, functioning as antigen-presenting cells. For example, fibrocytes are reported to act as potent antigen presenting cells [89,90].

CD34^+^ fibroblasts are reported to reside in the mammary glands, submandibular glands, and thyroid, probably acting in immune surveillance [91,92,93], and they play a pathological role in the lacrimal glands and conjunctiva in cGVHD-related dry eye disease [22,67,69]. Fibroblasts or mesenchymal cells in the stroma may play a role in maintaining the homeostasis of the ocular surface, including the lacrimal gland microenvironment, under healthy conditions, and they act as a regenerative or pathogenic trigger of inflamed tissue [94]. Mesenchymal stromal cells have been shown to be sensors and switches in inflammation [94]. In cGVHD-related dry eye disease, freshly isolated fibroblasts/fibrocytes may be partially derived from the bone marrow and activated to trigger immune-mediated fibrosis in cGVHD by interacting with immune cells, including macrophages, T cells, and B cells [67,69]. The fibrotic process is apparently accelerated, and the resultant excessive fibrosis leads to functional dysfunction in target organs, including the lacrimal glands and conjunctiva [67]. It is likely that the prolonged fibrotic environment in chronic inflammatory lacrimal gland and conjunctiva promotes the recruitment and mobilization of donor-derived fibroblast precursors into inflammatory milieu, which may have unlimited growth potential or may be constantly migrated from circulation in chronic GVHD [67].

In contrast, recent evidence has shown that cultured mesenchymal stem cells (MSCs) are useful for modulating and suppressing GVHD and have been used to treat steroid-refractory cGVHD and regenerate or suppress ocular GVHD in animal models [95,96]. This controversy is due to differences in the use of cultured mesenchymal stem cells or freshly isolated mesenchymal cells, the species of origin, the preparation methods, or the origin of the mesenchymal stem cells used. It may be useful to revisit freshly isolated mesenchymal stem cells whether they are linked to pathogenetic properties in GVHD-related dry eye disease [69]. An evaluation of prospective transplantation of freshly purified bone marrow mesenchymal/stromal cells (BMSCs) and hematopoietic stem cells into BALB/c-RAG2KO mice suggested that transplanted minor antigen-mismatched MHC-compatible BMSCs interact with residual host T cells to induce the autoimmune phenotype observed in fibrosis associated with the sclerodermatous mouse model [69]. While the driver antigen remains to be elucidated, this study suggests that the accidental recognition of self-minor antigens on MHC class II^+^ bone marrow stromal cells may be involved in pathogenic fibrosis observed in autoimmune diseases, including ocular GVHD [22,67,69].

### 3.2. Epithelial–Mesenchymal Transition (EMT)

Among the fibrotic processes that could account for cGVHD-related dry eye disease, EMT is a possible candidate for the process because the ocular surface epithelium loses the capability to secrete mucins when the epithelial cells undergo the transition to a mesenchymal phenotype [23]. Several studies have shown that EMT contributes to various fibrotic diseases of the kidneys, lungs, and liver and the ocular surface epithelium [97,98,99] and retinal pigment epithelium [100] in the eye. For example, 40% of fibroblasts in kidney fibrosis arise from epithelial cells via local EMT triggered by inflammatory stress, and 15% of fibroblasts are derived from the bone marrow under inflammatory stress [101]. EMT is characterized by the loss of apical/basal cell polarity and loss of cell-to-cell adhesions, which is followed by the acquisition of a mesenchymal phenotype that promotes migration and invasion capabilities and the expression of mesenchymal markers. EMT is triggered by various stimuli, including irradiation [102]; hypoxia [103]; reactive oxygen species [104]; inflammatory cytokines, such as TGF-β and fibroblast growth factor [105]; disruption of the basal lamina; and exposure of the cytoplasm to the extracellular matrix [106]. These triggers of EMT also cooperate with the pathogenesis of cGVHD after HSCT. Total body irradiation and migrating inflammatory cells generate substantial proinflammatory cytokines [3]. The “cytokine storm” influences T cells in the recipient microenvironment, prompting them to attack host antigens. In addition, reactive oxygen species-mediated lacrimal gland injury has been reported in the bone marrow transplant [107] and other target organs [108] in mice as possible triggers for EMT.

Notably, the rearrangement of cytoskeletal actin filaments is necessary for EMT, but an intact cytoskeleton is required to guide secretary vesicles to the ocular surface and to generate microvilli. Since cGVHD may trigger EMT in the ocular epithelium, the conjunctival microvilli may be abnormal and unable to secrete membrane-spanning mucins [49]. In ocular cGVHD, the production of both gel-forming mucins including MUC5AC and membrane-spanning mucin including MUC1, MUC4, and MUC16 is highly reduced [49]. It is likely that cytotoxic T cells cause the basement membrane of the conjunctival and lacrimal gland epithelium to break down [22,23,49], allowing direct interactions between the cytoskeleton of epithelial cells and stromal extracellular matrix components [106], which may trigger EMT in HSCT recipients.

## 4. Cellular Senescence in Ocular GVHD 

**Figure 2:**
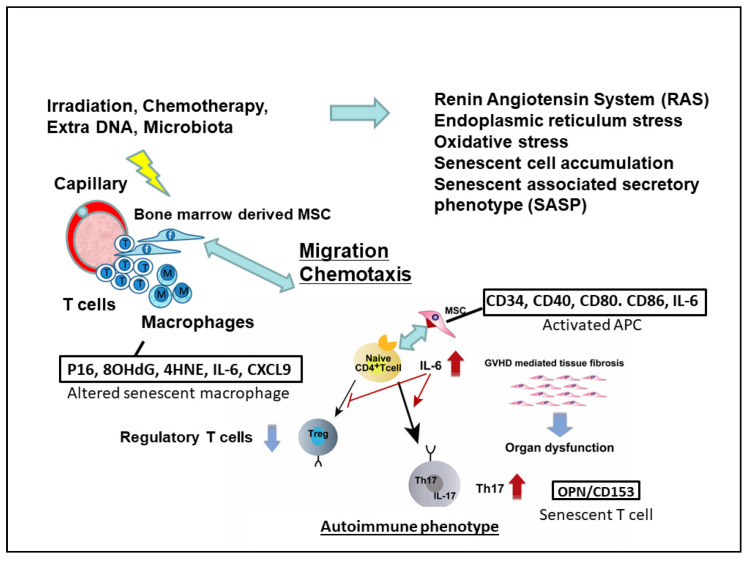
Migration of senescent macrophages play a key role in ocular GVHD. A pro-inflammatory state related to the conditioning regimen, and previous acute GVHD, may lead to an accumulation of cell debris, resulting in the activation of RAS, ER stress, oxidative stress, and senescent cell involving SASP accumulation on the ocular surface and in the lacrimal gland. 8-OHdG; 8-hydroxy-2′-deoxyguanosine, 4-HNE; 4-hydroxy-2-nonenal, HEL; hexonoyl lesion, IL-6, interleukine-6, CXCL9; C-X-C motif ligand 9, OPN; osteopontin, CD; clustered of differentiation.

Aging is commonly defined as the accumulation of diverse deleterious changes in cells and tissues with advancing age that are responsible for increased risks of disease [109]. In addition, cellular senescence involves genomic instability, telomere loss, oxidative damage, genetic programming, and cell death [109]. The activation of reactive oxygen species [107], renin angiotensin system [110], vascular adhesion protein-1 [111], and endoplasmic reticulum stress [112] together may contribute to stress-induced senescence in ocular GVHD. The pathogenesis of ocular GVHD and stress-induced cellular senescence are potentially associated through the senescence-associated secretory phenotype (SASP). Senescent cells produce cytokines and chemokines in terms of SASP [113], such as IL-6 and CXCL9 in GVHD lacrimal gland in mice [83] (Figure 2).

### 4.1. Oxidative Stress

Oxidative damage of immune cells may be the earliest sign of immune aging. Recently, macrophages were reported to be promoters of age-related diseases such as macular degeneration [114]. Other studies have shown that oxidative stress in mitochondria can activate ROS, leading to inflammasome activation and the production of pro-inflammatory cytokines [115], resulting in inflammation and age-related findings. In previous reports on immune-mediated dry eye disease in animal models, mitochondrial alterations were found to be central to the disease process [116,117]. The mitochondria contribute to the process of inflammation, and pro-inflammatory soluble factors apparently alter mitochondrial function to amplify their effects. The elevation of mitochondrial oxidative stress results in a vicious inflammatory cycle that exacerbates immune-mediated dry eye disease [118,119]. ROS are elevated in macrophages infiltrated in the lacrimal gland affected by cGVHD in a sclerodermatous mouse model [107]. Oxidative stress markers, including 8-hydroxy-2′-deoxyguanosine (8-OHdG), 4-hydroxy-2-nonenal (4-HNE), and hexonoyl lesion (HEL), and other aging markers, including p16 and p38, were detected in the infiltrating mononuclear cells, mainly the macrophages and endothelium in capillaries in cGVHD and aged mice but not in a syngeneic model or young mice [107].

### 4.2. Tissue Renin–Angiotensin System (RAS) 

The RAS was originally reported to be a blood pressure regulator. Besides systemic RAS, local tissue RAS activation is involved in the innate and adaptive immune systems and fibrosis. Yaguchi, S et al. proved that an angiotensin II (Ang II) type 1 receptor antagonist attenuate lacrimal gland, lung, and liver fibrosis in a murine model of cGVHD [110]. Ang II is reported to be a pro-inflammatory molecule and possible profibrotic molecule that affects progressive damage to the lacrimal glands and ocular surface homeostasis in ocular GVHD. It is reported that the binding of Ang II to the angiotensin type I receptor generates an intracellular free radical that plays a crucial role in tissue damage by accelerating mitochondrial dysfunction [120]. Those findings suggest that tissue RAS is related to stress-induced senescence in ocular GVHD. Blocking Ang II signaling protects against neurodegenerative processes and promotes longevity in an animal model, suggesting targeting Ang II signaling as a therapeutic strategy in inflammatory diseases, including GVHD-related dry eye disease and aging-related changes [121].

### 4.3. Endoplasmic Reticulum (ER) Stress

Cellular senescence has been reported to be detrimentally involved in ocular cGVHD, and cGVHD can be regarded as an age-associated disease [107]. Telomere shortening in donor hematopoietic stem cells (HSCs) after HSCT [122] suggests that donor immune cells as well as engrafted recipient cells are more senescent in cGVHD under an inflammatory milieu. Previous studies indicate that ER stress plays a role in chronic inflammation and age-related diseases [123,124]. The ER is a cellular organelle essential for proper cell function. When proteins are synthesized in the ER, they need to be folded correctly, and ER chaperones assist in protein folding [125]. However, hypoxia, calcium ion depletion, oxidative injury, viral infections, and inflammatory cytokines prevent the ER from performing its role in normal protein folding [126]. When unfolded and misfolded proteins accumulate in the ER, they cause ER stress, and the following three transmembrane proteins are consequently activated to initiate the unfolded protein response (UPR): inositol requiring (IRE) 1α, PKR-like ER kinase (PERK), and activating transcription factor (ATF) 6α [125]. However, if the UPR is prolonged or unsuccessful, inflammatory and apoptotic pathways are activated [125]. Therefore, an impaired UPR l results in the expression of (1) the pro-inflammatory molecule thioredoxin interaction protein (TXNIP) and transcription factor NF-κB and (2) the apoptotic protein C/EBP homologous protein (CHOP) [112,127].

Mukai S, et al. revealed that the ER stress is elevated in target organs of GVHD including the lacrimal gland and conjunctiva, and ER stress suppressor as well as anti-aging medicine, 4-phenyl butyric acid, can effectively reduce GVHD-related inflammation and fibrosis in targeted organs [112]. Those findings suggest that the pathogenic process of GVHD has aspects of stress-induced senescence and is linked to the underlying mechanism of the pathogenic process of ocular GVHD. Inhibition of the ER stress pathway may be a therapeutic target for cGVHD-related dry eye disease.

### 4.4. Vascular Adhesion Protein-1

With respect to the aberrant accumulation of inflammatory cells, the overexpression of vascular adhesion protein-1 (VAP-1) has been reported to be implicated in inflammatory diseases [128]. VAP-1 is an endothelial surface glycoprotein that mediates the migration of leukocytes from the bloodstream to tissues [129]. VAP-1 has the following two crucial portions that assist leukocytes in migrating to tissues: a distal adhesion domain and a catalytic amine oxidase region [130]. When immune cells infiltrate sites of inflammation, an amino group in each leukocyte attacks the carbonyl group in VAP-1 [130]. The primary amine is subsequently converted to the corresponding aldehyde, and this catalytic transformation enables inflammatory cells to extravasate into tissues through the blood vessel. Aldehyde is cytotoxic and related to oxidative stress, which plays a role in stress-induced senescence in some way in ocular GVHD. Based on these reports, Mukai S, et al. studied the following: (1) the prevention of immune cell migration to tissues alleviates acute GVHD (aGVHD) and cGVHD, (2) the increased expression of VAP-1 is involved in the pathogenesis of both types of GVHD, and (3) the inhibition of VAP-1 could be an efficacious treatment for the two types of GVHD in a mouse model. The protection of goblet cells through the injection of a VAP-1 inhibitor effectively improves the number of goblet cells in the intestine and conjunctiva in comparison with vehicle-treated GVHD mice.

### 4.5. Stress-Induced Senescence in Ocular GVHD

Yamane M, et al. [83] showed that the senescent-associated secretory phenotype (SASP) of macrophages, T cells, and fibroblast from lacrimal gland in GVHD mice is elevated and plays an indispensable role for chronic inflammation and fibrosis in ocular GVHD. The authors hypothesized that several stressors arrest the cell cycle, and macrophages exposed to severe damage undergo senescence and produce the SASP factors IL-6 and CXCL9 upon interaction with CD4^+^osteopontin (OPN)^+^CD153^+^ senescent T cells. Then, IL-6, a major driver of the SASP, reinforces macrophage senescence in an autocrine manner. In addition, CXCL9 facilitates the recruitment of neighboring T cells to the microenvironment early after onset [131]. T cell senescence after HSCT was recently reported in an acute GVHD animal model. CD4^+^OPN^+^CD153^+^ senescent T cells may accelerate stress-induced senescence as a late complication of cGVHD. Moreover, IL-6 may induce senescence in a subpopulation of fibroblasts. In addition, OPN, another SASP factor, promotes EMT in cGVHD lacrimal glands. As a result, a subset of senescent/mature fibroblasts synthesize abnormal collagens. Fibroblasts affected by macrophages and T cells also synthesize excessive abnormal collagens and extracellular matrix components [15,70], leading to inflammation and abnormal fibrosis in cGVHD lacrimal glands. Collectively, senescent cells with the SASP or cells with stress-induced senescent features contribute to the pathogenesis of cGVHD in the lacrimal glands, although immunological inflammation is the major underlying mechanism. Inhibiting the SASP induced by senescent cells, using senolytic reagents may be a new clinically translatable strategy for attenuating the effects of cGVHD on the lacrimal glands and other target organs.

## 5. Treatment

According to the basic mechanism of chronic ocular GVHD, an anti-inflammatory, anti-fibrotic, and anti-aging approach will be required to add or radical treatment in addition to the common approaches including moisture, lubricant, epithelial support, and surgical treatments (Table 1) [3,4,6,45,132,133,134,135].

A specific prophylaxis and/or treatment of ocular GVHD may be utilized including decontamination such as doxycycline [6,143,144,165,166,167]. Pooled human immunoglobulin eye drops have been reported to be effective for ocular GVHD [32]. It can be also used for prophylaxis and/or the treatment of ocular GVHD. At the basic study stage, gentamicin is a possible candidate for the prophylaxis of ocular GVHD [145].

The recommended treatments of ocular GVHD in the inflammatory phase are as follows. Topical preservative-free corticosteroids [143], rebamipide, cyclosporine A [143], and tacrolimus [144,146] for ocular surface inflammation have been shown to be effective. Low-dose topical corticosteroid is shown to have reduced efficacy on ocular GVHD [168]. One of the biological agents, lifitegrast (Xiidra), a topical integrin antagonist, was well-tolerated and led to an improvement in symptoms of KCS in eight (44%) patients [169]. Due to multifactorial aspects of this disease, careful follow up on unexpected events is required [170]. The other biological agent, Anakinra (IL-1Ra antagonist), by suppressing IL-1-mediated inflammation by competitively inhibiting the binding of IL-1α and IL-1β to IL-1 receptor I, may be beneficial as a therapeutic option for patients with dry eye disease [171], and it might have a beneficial effect on ocular GVHD. Tranilast is N-(30, 40-dimethoxycinnamoyl)-anthranilic acid and an analog of a tryptophan metabolite [172]. It has an inhibition of the inflammatory effect by suppressing the recruitment of T cells by the inhibition of CXCL9 expression, an anti-oxidative stress effect through the inhibition of thiorexin-interacting protein (TXNIP) [173], and an anti-fibrotic effect by inhibition of fibroblast activation. Topical tranilast has been reported in a small number of cGVHD-related dry eye patients [174].

As epithelial support, autologous serum has been increased to be used as eye drops for ocular GVHD because it contains the vitamins, several growth factors, and fibronectin that are crucial for ocular surface integrity [134,148,149,175,176]. Cord blood serum [150,151], or platelet lysate eye drops [152,153] also have been reported to show benefits for ocular GVHD patients. Recently, the detection of systemic immunosuppressants in serum has been reported and benefits from using a topical serum eye drop on ocular GVHD [177].

Surgical and other treatments are shown in Table 1 and details are described in cited references.

As a consequence of the ocular condition during systemic therapy, the average response rates of ocular GVHD are 43% for extracorporeal photopheresis [178,179], 31% for rituximab (anti-CD20 antibody), 60% for sirolimus, and 33% for mycophenolate mofetil in corticosteroid-refractory GVHD [6].

It has been shown that IL-6 is elevated early in the course of ocular GVHD in animal models [69,83] and humans [25,26,28,83,180,181]. Systemic tocilizumab [182,183] and sarilumab for rheumatoid arthritis [184] that impact the IL-6 pathway may be beneficial to treat or prevent ocular GVHD. Other biologics including janus kinase (JAK) combined with spleen tyrosine kinase (SYK) have been reported to be well tolerated, suggesting efficacy for ocular GVHD patients as a pilot study [185]. It will be benefit the severe ocular GVHD patients if the biological reagents used both systemic and topical eye drops or ointments as well.

In basic research, ATR type I antagonist [110], VAP-1 inhibitor [111], phenyl butyric acid [112], tranilast [186], heavy chain-hyaluronan/pentraxin 3 (HC-HA/PTX3) [187], ABT-263, a senolytic agent that removes senescent cells, an anti-IL-6 blocking antibody for SASP inhibition [83], and vitamin A-coupled liposomes containing HSP4 siRNA reversed the changes seen in ocular GVHD [162]. Entospletinib (ENTO), small molecule inhibitors of lymphocyte signaling, and a second-generation highly selective SYK inhibitor [147] have been reported to reduce inflammation and its by-product and/or fibrosis in ocular as wells systemic GVHD mice. Pooled human immunoglobulin eye drops have potential as a new candidate in treating ocular GVHD [32]. Creating innovative therapies for ocular GVHD is necessary to treat this intractable ocular disease. Ocular GVHD must be recognized and treated early in the course to prevent the irreversible progression and damage of ocular tissue using these strategies. If the ocular GVHD is found at a later stage, those therapies should be started as early as possible to slow or prevent the further development of this disease.

## 6. Future Directions

Future therapeutic goals include regeneration of the dysfunctional ocular surface, lacrimal glands and meibomian glands. Broad-based translational research that extends from bench to bedside and makes use of clinical results (bedside to bench) will augment current analyses of the mechanisms underlying dry eye disease and will lead to the development of new anti-inflammatory, anti-fibrotic, and anti-senescent approaches or other specific interventions based on these mechanisms.

## 7. Patents

Y.O. has a patent in Japan (Patent No. 4966019; Name; Topical application and oral intake of tranilast for the treatment of chronic GVHD-related dry eye disease) and Y.O and K.T. have patent application number JP 2017-018643 published as JPA2017-178922, application number JP2018-510646 published as WO2017/175808, and application number JP 2019-004730 published as JPA2020-111548. Y.K. reports no conflicts of interest related to this study.

## Figures and Tables

**Table 1:** Possible treatments/prophylaxis of ocular cGVHD at present and in the future [3,6,12,132,133,134,136,137].

Strategy	Medicine or Device
Retention of tear fluid	Preservative-free artificial tear, Diquafosol [138], Punctal plug [139], Surgical punctal occlusion [140], Oral muscarinic agonists (pilocarpine [141], cevimeline [12]),
Mucin producing, tear film stabilization	Diquafosol [138], Rebamipide [142]
Reduction of inflammation and by-products	Preservative-free corticosteroid [143], Cyclosporin [143], Tacrolimus [144,145,146], Tranilast, IL-1 receptor inhibitor, Lifitegrast, DNase [82], immunoglobulin [32], (Basic study; Heparin [81], Systemic vascular adhesion protein-1 inhibitor [111], Phenyl butyric acid [112], Senolytic agent [83,112]. Small molecule inhibitors of lymphocyte signaling [147], Anti-IL-6 receptor antagonist [83])
Epithelial support	Hyaluronic acid, Autologeous sera [148,149], Cord blood sera [150,151], Platelet lysate [152,153], Amniotic membrane transplantation [4,154,155,156,157], Cultured epithelial cell transplantation, Medical use contact lenses [158,159,160,161].
Prevention of tear evaporation	Moisture goggle, Tetracycline, Warm compression, Lid hygiene.
Inhibition of refractory ocular and systemic GVHD	Systemic administration (Tacrolimus, Cyclosporin, Corticosteroid, Extracorporeal photopheresis, Rituximab, Sirolimus (Rapamycin), Mycophenolate mofetil) [6]
Reduction of fibrosis	(Basic study, Angiotensin type I receptor antagonist [110], Vitamin A-coupled liposomes containing HSP47 siRNA [162])
Supportive care	Moisture goggle, Prevention of infection (Doxycycline, Minocycline) [3], Maintenance of environmental factor [163]
Other surgical treatment [5,132]	Epithelial cell debridement [5] Surgical correction of entropion [132,164] Partial tarsorrhaphy Fornix reconstruction Penetrating keratoplasty Lamellar keratoplasty
